# Distinct Clinicopathological Features of HER2‐Negative, HER2‐Low, and HER2‐Overexpressing Urothelial Carcinoma in a Large Chinese Cohort

**DOI:** 10.1002/cam4.71289

**Published:** 2025-10-10

**Authors:** Shanshan Wang, Dingwei Ye, Li Yang, Fan Cheng, Tiejun Yang, Xiaoping Zhang, Zhixian Yu, Qingyun Zhang, Yong Yang

**Affiliations:** ^1^ Department of Urology Fudan University Shanghai Cancer Center Shanghai China; ^2^ Department of Oncology, Shanghai Medical College Fudan University Shanghai China; ^3^ Department of Urology The Second Hospital of Lanzhou University Lanzhou China; ^4^ Department of Urology Renmin Hospital of Wuhan University (Hubei General Hospital) Wuhan China; ^5^ Department of Urology Henan Cancer Hospital Zhengzhou China; ^6^ Department of Urology, Union Hospital, Tongji Medical College Huazhong University of Science and Technology Wuhan China; ^7^ Department of Urology The First Affiliated Hospital of Wenzhou Medical University Wenzhou China; ^8^ Department of Urology The Affiliated Tumor Hospital of Guangxi Medical University Nanning China; ^9^ Department of Urology Peking University Cancer Hospital Beijing China

**Keywords:** china, ErbB‐2, immunohistochemistry, receptor, urothelial carcinoma

## Abstract

**Purpose:**

To investigate the expression patterns of Human Epidermal Growth Factor Receptor 2 (HER2) and their clinicopathological associations across the full spectrum (negative, low, and overexpression) in a large cohort of Chinese urothelial carcinoma (UC) patients.

**Materials and Methods:**

A multicenter registry study (April 2023–March 2024) across eight Chinese tertiary hospitals included 1054 UC patients. Demographic, clinical, and pathological data were analyzed to identify factors associated with different HER2 expression levels (IHC 0 vs. 1+ vs. 2+/3+). A subset of patients was evaluated for additional IHC markers (e.g., CK20, GATA3, P16, Uroplakin3, Ki‐67).

**Results:**

Of 1054 patients, 18.6% were HER2‐negative (IHC 0), 23.0% were HER2‐low (IHC 1+), and 58.4% exhibited HER2 overexpression (IHC 2+/3+). Increasing HER2 expression was significantly associated with bladder tumor location (63.1% in IHC 2+/3+, *p* < 0.001), infiltrative tumors (61.1% in IHC 2+/3+, *p* < 0.001), and high‐grade tumors (62.5% in IHC 2+/3+, *p* < 0.001). In a sub‐analysis comparing HER2‐low (1+) and HER2‐overexpressing (2+/3+) groups, multivariable logistic regression confirmed bladder primary site (OR = 1.783, *p* = 0.001), infiltrative status (OR = 1.492, *p* = 0.027), and high‐grade differentiation (OR = 1.918, *p* = 0.001) as independent predictors of HER2 overexpression, though the model's predictive ability was modest (AUC = 0.64). Expression of CK20, GATA3, P16, and Uroplakin3 also differed significantly between HER2‐negative and HER2‐positive groups.

**Conclusions:**

This study delineates distinct clinicopathological profiles for HER2‐negative, HER2‐low, and HER2‐overexpressing UC in Chinese patients. These findings provide a crucial evidence base for refining personalized treatment strategies, particularly for HER2‐targeted therapies like antibody‐drug conjugates (ADCs), across the entire spectrum of HER2 expression.

## 1 Introduction

Urothelial carcinoma (UC) represents a significant global health burden, ranking among the most common malignancies of the genitourinary system, with bladder cancer constituting the majority (80%–90%) of cases [1]. The incidence and mortality of UC are rising, notably in China, where GLOBOCAN 2020 reported 85,000 new bladder cancer cases and 39,000 deaths [2]. UC is a heterogeneous disease characterized by high malignancy, recurrence rates, and resistance to chemotherapy. Despite advancements in oncology, progress in developing effective therapies for advanced UC has been relatively slow, leading to poor prognoses for many patients [3].

The human epidermal growth factor receptor 2 (HER2) has emerged as a significant factor in UC progression, with its overexpression linked to poor outcomes. HER2 expression is commonly assessed by immunohistochemistry (IHC) and is categorized based on staining intensity into negative (0), low expression (1+), and overexpression (2+/3+) [4]. While HER2 overexpression has been a target for therapies, conventional monoclonal antibodies and small‐molecule inhibitors have not consistently demonstrated clinical benefits in HER2‐overexpressing UC [4].

Recent advances in antibody‐drug conjugates (ADCs) targeting HER2 have shown significant clinical promise in UC [5]. These agents, which combine HER2‐specific antibodies with potent cytotoxic drugs, have improved outcomes, particularly when used with immune checkpoint inhibitors for HER2‐overexpressing advanced UC [6]. For instance, clinical trials combining disitamab vedotin with tislelizumab have reported encouraging objective response rates (ORR) of 62.5% and disease control rates (DCR) of 87.5% [7]. These findings underscore the importance of accurately characterizing HER2 expression to identify patients most likely to benefit from these emerging therapies [8, 9].

Our prior multicenter study provided preliminary insights into HER2 prevalence in Chinese UC patients, indicating that a history of smoking, lipid metabolism diseases, tumor location, differentiation status, and tumor stage might be the factors affecting HER2 expression [10]. However, that work did not fully explore the nuanced differences across the entire spectrum of HER2 expression (0, 1+, and 2+/3+) or the role of additional IHC markers. This study addresses these critical gaps by conducting a comprehensive analysis of 1054 patients, comparing clinicopathological characteristics across HER2‐negative, HER2‐low, and HER2‐overexpressing groups. We also evaluate the expression of other IHC markers in relation to HER2 status. These analyses provide novel insights into the molecular heterogeneity of UC, enhancing the global understanding of HER2 in this disease and guiding the development of personalized treatment strategies, especially in the context of emerging HER2‐targeted ADCs.

## 2 Methods

### 2.1 Study Rationale

Building on our previous HER2 prevalence survey, this study expands the sample size to comprehensively compare clinicopathological features across HER2‐negative (0), HER2‐low (1+), and HER2‐overexpressing (2+/3+) UC. It also incorporates a broad IHC marker panel, a novel approach in large Chinese cohorts.

### 2.2 Study Design

We analyzed data from 1054 UC patients from a multicenter registry across eight Chinese tertiary hospitals (April 2023–March 2024). Inclusion criteria were: age > 18, histopathologically confirmed UC, and complete IHC HER2 results for the primary tumor. Exclusion criteria included tumors at other sites or conditions deemed unsuitable by physicians. The study was approved by the Ethics Committee of Fudan University Shanghai Cancer Center (Approval No.: 2301268–12) and registered with the Chinese Clinical Trial Registry (ChiCTR2300069746).

### 2.3 HER2 IHC Testing and Other Markers

HER2 expression in primary UC tumors was assessed by IHC in the pathology departments of each participating center. While specific antibodies and platforms varied between centers, a common example includes the Ventana PATHWAY anti‐HER‐2/neu (4B5) rabbit monoclonal antibody. All centers adhered to the 2021 Clinical Pathology Expert Consensus on HER2 Testing in Chinese Urothelial Carcinoma^4^, which defined the standard for IHC scoring. HER2 expression was scored as 0 (negative), 1+ (low/weak expression), 2+ (equivocal overexpression), or 3+ (strong overexpression). For analysis, 2+ and 3+ were combined into a single “overexpression” group. Other IHC markers, such as CK7, CK20, GATA3, and p63, were tested using commercially available antibodies, following manufacturers' protocols.

### 2.4 Data Sources

Data were collected from hospital medical record systems, including demographic information, medical history, ECOG score, pathological examination results (type, grade, TNM stage, HER2 status, and other IHC markers), and treatment plans. This study focused on HER2 expression patterns without survival analysis, as outcome data collection is ongoing.

### 2.5 Statistical Analysis

Statistical analyses were performed using SAS 9.4. Continuous variables were reported as mean ± SD or median [IQR] and analyzed using t‐tests or ANOVA. Categorical variables were compared across the three HER2 groups (0, 1+, 2+/3+) using chi‐square or Fisher's exact tests (*p* < 0.05 for significance). A binary logistic regression was performed as a sub‐analysis to identify factors associated with HER2 overexpression (2+/3+) versus low expression (1+), including variables with *p* < 0.2 in univariate analysis. Predictive ability was assessed via receiver operating characteristic (ROC) curve analysis.

## 3 Results

### 3.1 Patient Characteristics

A total of 1054 patients were included in the final analysis. The mean age was 66.8 ± 10.5 years, and 78.5% were male. The cohort comprised 76.6% (*n* = 807) bladder UC and 23.4% (*n* = 247) upper tract urothelial carcinoma (UTUC). Overall, 4.8% (50/1043) of patients presented with distant metastases at diagnosis, primarily to lymph nodes (58.3%), bone (27.1%), and lung (20.8%). Regarding HER2 expression, 18.6% (*n* = 196) were HER2‐negative (IHC 0), 23.0% (*n* = 242) were HER2‐low (IHC 1+), and 58.4% (*n* = 616) had HER2 overexpression (IHC 2+/3+).

### 3.2 Association of HER2 Expression With Clinicopathological Features

We observed significant associations between HER2 expression levels and several key clinicopathological features (Table 1). A stepwise increase in HER2 expression was correlated with: (1) Primary Tumor Site: Bladder origin was more frequent with higher HER2 expression (62.2% for IHC 0, 72.7% for IHC 1+, and 82.6% for IHC 2+/3+; *p* < 0.001); (2) Infiltrative Status: The proportion of infiltrative tumors increased with HER2 expression levels (*p* < 0.001); (3) Histological Grade: High‐grade (moderately/poorly differentiated) tumors were more common in HER2‐overexpressing patients compared to HER2‐negative and HER2‐low groups (*p* < 0.001); (4) Clinical Stage: There was a significant difference in clinical stage distribution for both bladder and upper tract cancers across the HER2 groups (*p* < 0.01).

**TABLE 1:** Clinicopathological Characteristics According to HER2 Expression Status (IHC 0 vs. 1+ vs. 2+/3+).

Variable	Total *n* (col%)	IHC 0 *n* (col%, row%)	IHC 1+ *n* (col%, row%)	IHC 2+/3+ *n* (col%, row%)	*P*‐value
*n*	1054 (100.0%)	196 (/, 18.6%)	242 (/, 23.0%)	616 (/, 58.4%)	0.097
Age (years)					
< 60	243 (23.1%)	54 (27.6%, 22.2%)	61 (25.2%, 25.1%)	128 (20.8%, 52.7%)	
≥ 60	811 (76.9%)	142 (72.4%, 17.5%)	181 (74.8%, 22.3%)	488 (79.2%, 60.2%)	
Gender					0.173
Male	827 (78.5%)	146 (74.5%, 17.7%)	186 (76.9%, 22.5%)	495 (80.4%, 59.9%)	
Female	227 (21.5%)	50 (25.5%, 22.0%)	56 (23.1%, 24.7%)	121 (19.6%, 53.3%)	
Smoking History					0.004
No	786 (74.6%)	164 (83.7%, 20.9%)	180 (74.4%, 22.9%)	442 (71.8%, 56.2%)	
Yes	268 (25.4%)	32 (16.3%, 11.9%)	62 (25.6%, 23.1%)	174 (28.2%, 64.9%)	
Primary Site					< 0.001
Bladder	807 (76.6%)	122 (62.2%, 15.1%)	176 (72.7%, 21.8%)	509 (82.6%, 63.1%)	
Upper Tract	247 (23.4%)	74 (37.8%, 30.0%)	66 (27.3%, 26.7%)	107 (17.4%, 43.3%)	
Infiltrating Status					< 0.001
Non‐infiltrating	406 (38.5%)	66 (33.7%, 16.3%)	120 (49.6%, 29.6%)	220 (35.7%, 54.2%)	
Infiltrating	648 (61.5%)	130 (66.3%, 20.1%)	122 (50.4%, 18.8%)	396 (64.3%, 61.1%)	
Histologic Grade					< 0.001
Low‐grade	141 (13.4%)	45 (23.0%, 31.9%)	51 (21.1%, 36.2%)	45 (7.3%, 31.9%)	
High‐grade	913 (86.6%)	151 (77.0%, 16.5%)	191 (78.9%, 20.9%)	571 (92.7%, 62.5%)	
Metastasis					0.576
No	993 (95.2%)	180 (93.8%, 18.1%)	229 (95.4%, 23.1%)	584 (95.6%, 58.8%)	
Yes	50 (4.8%)	12 (6.2%, 24.0%)	11 (4.6%, 22.0%)	27 (4.4%, 54.0%)	

### 3.3 Subgroup Analysis on the Subjects With HER2 (1+) and (2+/3+) Urothelial Carcinoma

In the observation of characteristic of HER2 (1+) and (2+/3+) UC patients, significant differences were observed between HER2 1+ and 2+/3+ groups for primary tumor site (*p* = 0.001), infiltration status (*p* < 0.001), and differentiation level (*p* < 0.001). Bladder tumors were more common in the HER2 2+/3+ group than upper tract tumors (63.1% vs. 43.3%, *p* = 0.001), as were infiltrative tumors (61.1% vs. 54.2% in Non‐infiltrating, *p* < 0.001) and high‐grade tumors (62.5% vs. 31.9% in low‐grade, *p* < 0.001). To test the robustness of these results, subgroups based on these factors were also tested, and some factors lost significance: primary site in the low‐grade subgroup (HER2 1+: 69 [84.1%], HER2 2+/3+: 102 [87.2%], *p* = 0.545), infiltration status in the same subgroup (*p* = 0.088), and differentiation level in the infiltrative subgroup (HER2 1+: 11 [8.9%], HER2 2+/3+: 27 [6.8%], *p* = 0.429) (Table 2).

**TABLE 2:** Characteristics of the subjects with HER2 (1+) and (2+/3+) urothelial carcinoma and subgroup analysis.

		All population			Bladder subgroup			Infiltrating subgroup			Low/High grade subgroup		
		HER2 (1+)	HER2 (2+/3+)	*P*	HER2 (1+)	HER2 (2+/3+)	*P*	HER2 (1+)	HER2 (2+/3+)	*P*	HER2 (1+)	HER2 (2+/3+)	*P*
		*n* = 242	*n* = 616		*n* = 176	*n* = 509		*n* = 123	*n* = 396		*n* = 82	*n* = 117	
Age	18–44	8 (3.3%)	13 (2.1%)	0.512	7 (4.0%)	12 (2.4%)	0.489	2 (1.6%)	6 (1.5%)	0.587	5 (6.1%)	7 (6.0%)	0.566
	45–59	54 (22.3%)	118 (19.2%)		38 (21.6%)	94 (18.5%)		28 (22.8%)	70 (17.7%)		16 (19.5%)	29 (24.8%)	
	60–74	129 (53.3%)	348 (56.5%)		95 (54.0%)	285 (56.0%)		64 (52.0%)	230 (58.1%)		50 (61.0%)	60 (51.3%)	
	> = 75	51 (21.1%)	137 (22.2%)		36 (20.5%)	118 (23.2%)		29 (23.6%)	90 (22.7%)		11 (13.4%)	21 (17.9%)	
BMI	Underweight	2 (0.8%)	16 (2.6%)	0.255	2 (1.1%)	13 (2.6%)	0.111	2 (1.6%)	13 (3.3%)	0.189	0 (0.0%)	0 (0.0%)	0.283
	Normal	141 (58.3%)	344 (55.8%)		108 (61.4%)	276 (54.2%)		72 (58.5%)	220 (55.6%)		51 (62.2%)	70 (59.8%)	
	Overweight	73 (30.2%)	203 (33.0%)		46 (26.1%)	175 (34.4%)		34 (27.6%)	134 (33.8%)		26 (31.7%)	32 (27.4%)	
	Obesity	26 (10.7%)	53 (8.6%)		20 (11.4%)	45 (8.8%)		15 (12.2%)	29 (7.3%)		5 (6.1%)	15 (12.8%)	
Gender	Male	186 (76.9%)	495 (80.4%)	0.255	141 (80.1%)	433 (85.1%)	0.124	95 (77.2%)	321 (81.1%)	0.353	64 (78.0%)	94 (80.3%)	0.694
	Female	56 (23.1%)	121 (19.6%)		35 (19.9%)	76 (14.9%)		28 (22.8%)	75 (18.9%)		18 (22.0%)	23 (19.7%)	
Ethnic Group	Han	235 (97.1%)	602 (97.7%)	0.597	170 (96.6%)	495 (97.2%)	0.655	122 (99.2%)	390 (98.5%)	0.555	80 (97.6%)	114 (97.4%)	0.956
	Minorities	7 (2.9%)	14 (2.3%)		6 (3.4%)	14 (2.8%)		1 (0.8%)	6 (1.5%)		2 (2.4%)	3 (2.6%)	
Smoking History	Non‐smoker	194 (80.2%)	494 (80.2%)	0.992	143 (81.3%)	400 (78.6%)	0.452	103 (83.7%)	323 (81.6%)	0.583	62 (75.6%)	87 (74.4%)	0.841
	Current/Previous Smoker	48 (19.8%)	122 (19.8%)		33 (18.8%)	109 (21.4%)		20 (16.3%)	73 (18.4%)		20 (24.4%)	30 (25.6%)	
Alcohol Taking History	Non‐alcoholic	221 (91.3%)	562 (91.2%)	0.967	162 (92.0%)	463 (91.0%)	0.661	113 (91.9%)	366 (92.4%)	0.840	72 (87.8%)	101 (86.3%)	0.760
	Current/Previous Alcoholic	21 (8.7%)	54 (8.8%)		14 (8.0%)	46 (9.0%)		10 (8.1%)	30 (7.6%)		10 (12.2%)	16 (13.7%)	
Primary Site of Tumor	Bladder	176 (72.7%)	509 (82.6%)	0.001	/	/	/	78 (63.4%)	310 (78.3%)	0.001	69 (84.1%)	102 (87.2%)	0.545
	Upper Tract	66 (27.3%)	107 (17.4%)		/	/	/	45 (36.6%)	86 (21.7%)		13 (15.9%)	15 (12.8%)	
Metastasis	No	229 (94.6%)	586 (95.1%)	0.909	166 (94.3%)	488 (95.9%)	0.537	114 (92.7%)	372 (93.9%)	0.611	79 (96.3%)	112 (95.7%)	0.499
	Yes	11 (4.5%)	27 (4.4%)		8 (4.5%)	18 (3.5%)		8 (6.5%)	21 (5.3%)		2 (2.4%)	5 (4.3%)	
Infiltrating	No	119 (49.2%)	220 (35.7%)	< 0.001	98 (55.7%)	199 (39.1%)	< 0.001	/	/	/	71 (86.6%)	90 (76.9%)	0.088
	Yes	123 (50.8%)	396 (64.3%)		78 (44.3%)	310 (60.9%)		/	/	/	11 (13.4%)	27 (23.1%)	
Level of Differentiation	Well Differentiated	82 (33.9%)	117 (19.0%)	< 0.001	69 (39.2%)	102 (20.0%)	< 0.001	11 (8.9%)	27 (6.8%)	0.429	/	/	/
	Moderately/Poorly Differentiated	160 (66.1%)	499 (81.0%)		107 (60.8%)	407 (80.0%)		112 (91.1%)	369 (93.2%)		/	/	/
Clinical Tumor Stage	Early/Intermediate	205 (84.7%)	499 (81.0%)	0.203	150 (85.2%)	435 (85.5%)	0.939	88 (71.5%)	290 (73.2%)	0.713	77 (93.9%)	106 (90.6%)	0.399
	Advanced	37 (15.3%)	117 (19.0%)		26 (14.8%)	74 (14.5%)		35 (28.5%)	106 (26.8%)		5 (6.1%)	11 (9.4%)	
History of Allergy	No	226 (93.4%)	573 (93.0%)	0.848	166 (94.3%)	476 (93.5%)	0.706	112 (91.1%)	364 (91.9%)	0.762	79 (96.3%)	110 (94.0%)	0.460
	Yes	16 (6.6%)	43 (7.0%)		10 (5.7%)	33 (6.5%)		11 (8.9%)	32 (8.1%)		3 (3.7%)	7 (6.0%)	
Treatment Strategy	None	5 (2.1%)	18 (2.9%)	0.239	4 (2.3%)	13 (2.6%)	0.305	4 (3.3%)	11 (2.8%)	0.270	1 (1.2%)	5 (4.3%)	0.322
	Surgical Resection	236 (97.5%)	595 (96.6%)		171 (97.2%)	494 (97.1%)		118 (95.9%)	383 (96.7%)		81 (98.8%)	111 (94.9%)	
	Radiation	1 (0.4%)	0 (0.0%)		1 (0.6%)	0 (0.0%)		1 (0.8%)	0 (0.0%)		0 (0.0%)	0 (0.0%)	
	Surgical Resection + Radiation	0 (0.0%)	3 (0.5%)		0 (0.0%)	2 (0.4%)		0 (0.0%)	2 (0.5%)		0 (0.0%)	1 (0.9%)	
Chronic Diseases	Hypertension	96 (39.7%)	251 (40.7%)	0.772	70 (39.8%)	213 (41.8%)	0.630	42 (34.1%)	160 (40.4%)	0.214	36 (43.9%)	42 (35.9%)	0.255
	Diabetes	42 (17.4%)	103 (16.7%)	0.823	29 (16.5%)	85 (16.7%)	0.946	23 (18.7%)	62 (15.7%)	0.426	11 (13.4%)	17 (14.5%)	0.824
	Cardiac/Cerebro Vascular Disease	44 (18.2%)	105 (17.0%)	0.693	29 (16.5%)	83 (16.3%)	0.958	19 (15.4%)	62 (15.7%)	0.955	17 (20.7%)	23 (19.7%)	0.852
	Dyslipidemia	7 (2.9%)	7 (1.1%)	0.068	6 (3.4%)	6 (1.2%)	0.052	2 (1.6%)	4 (1.0%)	0.577	3 (3.7%)	1 (0.9%)	0.165

### 3.4 Logistic Regression and ROC Analysis for HER2 Overexpression

**FIGURE 1:**
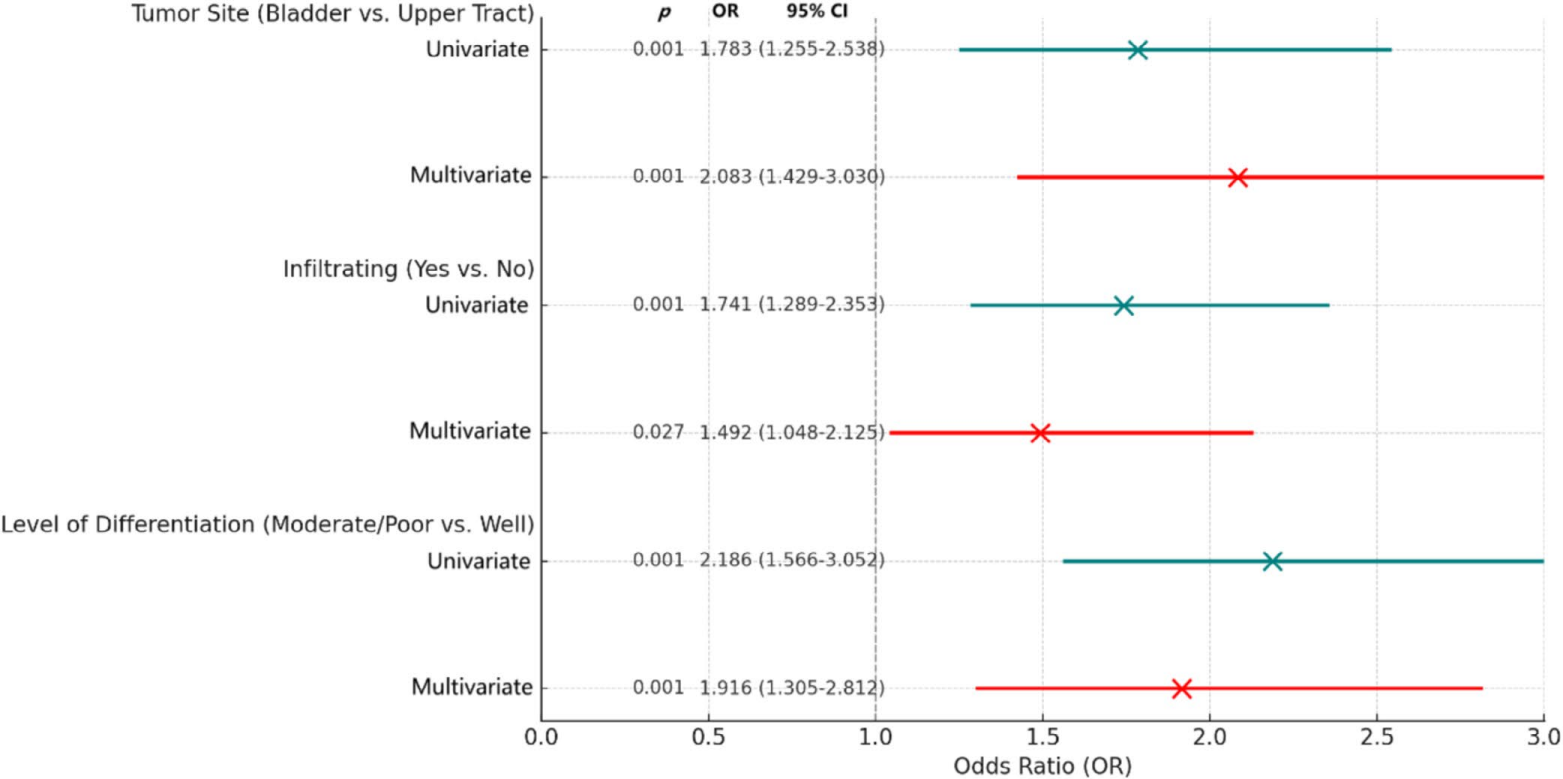
Forest plot for Univariate/multivariable Logistic Regressions. HER2 (2+/3+) vs. (1+) as the dependent variable. Adjusted for gender and smoking history in multivariable analysis.

**FIGURE 2:**
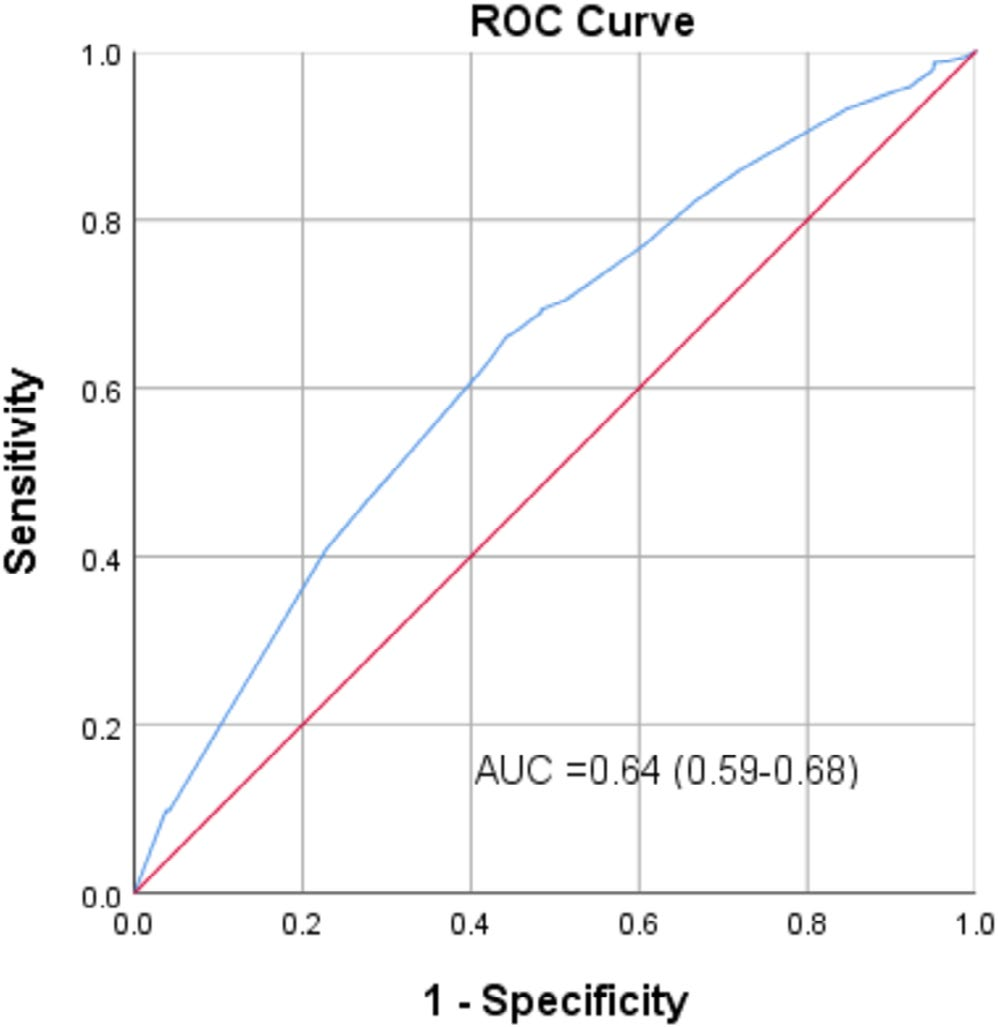
ROC of three factors for prediction of HER (2+/3+). ROC of three factors for prediction of HER (2+/3+), with AUC of 0.64 (95% CI: 0.59–0.88).

In a sub‐analysis comparing HER2‐low (1+) versus HER2‐overexpressing (2+/3+) patients, univariate analysis identified tumor site, infiltrating status, and histologic grade as significant factors. In the multivariable analysis, adjusted for gender and smoking history, all three remained independent predictors of HER2 overexpression: bladder primary site (OR = 1.783, *p* = 0.001), infiltrating status (OR = 1.492, *p* = 0.027), and high‐grade (OR = 1.918, *p* = 0.001) (Figure 1). This model was adjusted for age and smoking status due to their clinical relevance and significance in univariate analysis. The ROC curve analysis for this model yielded an AUC of 0.64 (95% CI: 0.59–0.68), indicating a modest ability to discriminate between HER2‐low and HER2‐overexpressing cases based on clinical features alone (Figure 2).

### 3.5 Association of Ancillary Immunohistochemistry Markers With HER2 Status

Additional IHC markers were analyzed exploratorily in an ancillary cohort of 378 patients from two centers (Fudan University Shanghai Cancer Center and Second Hospital Affiliated to Lanzhou University). Due to limited sample size and center representation, these exploratory results require cautious interpretation. Tested markers included CK7, CK20, GATA3, P53, CK5/6, Ki‐67, TRPS1, Syn, CDX2‐88, INSM1, P16, PD‐L1 22C3, AE1/AE3, P63, CgA, CA9, CD117, p504s, Vimentin, TFE3, CD10, P40, PSA, WT1, Uroplakin, Claudin18, cyclin D1, CK14, PSAP, SATB2, EGFR, E‐Cad, ERG, CD44, PSMA, Villin, CDX‐2, beta‐catenin, and CKp. Markers with test rates lower than 10% were excluded from the analysis. IHC markers which were included in this analysis were: Ki‐67, GATA3, CK20, CK7, P63, Uroplakin3, AR, P53, CK5/6, P16, TRPS1, INSM1, and CDX2. Compared to HER2‐negative (IHC 0) tumors, HER2‐positive (IHC 1+/2+/3+) tumors demonstrated significantly higher rates of positivity for CK20 (77.7% vs. 45.7%, *p* < 0.001), GATA3 (97.7% vs. 91.7%, *p* = 0.009), P16 (67.0% vs. 46.7%, *p* = 0.042), and Uroplakin3 (53.3% vs. 30.9%, *p* = 0.005). Furthermore, the proliferation index Ki‐67 was also significantly elevated in the HER2‐positive group (median 40% vs. 30%, *p* = 0.013). No statistically significant differences were observed for other markers, including CK7, P53, P63, and androgen receptor (AR), between the HER2‐negative and positive groups (Table 3). In addition, we explored the multi‐positive patterns of IHC markers; exploratory analysis of multiple positive IHC marker patterns in 378 patients revealed common combinations, such as CK7 + CK20 + CK5/6 + P63 + Uroplakin3 (8.2%) (Supplementary Table S1).

**TABLE 3:** IHC markers tested in UC patients, test rate and comparison of positive rate between HER2 status (0+ vs. 1+/2+/3+).

IHC markers	Test rate	Result	Whole registry	HER2 (−)	HER2 (1+/2+/3+)	*P*
CK7	85.7%	−	16 (4.9%)	6 (6.7%)	10 (4.3%)	0.373
		+	308 (95.1%)	84 (93.3%)	224 (95.7%)	
CK20	94.2%	−	109 (30.6%)	50 (54.3%)	59 (22.3%)	< 0.001
		+	247 (69.4%)	42 (45.7%)	205 (77.7%)	
GATA3	94.7%	−	14 (3.9%)	8 (8.3%)	6 (2.3%)	0.009
		+	344 (96.1%)	88 (91.7%)	256 (97.7%)	
P53	41.0%	−	14 (9.0%)	4 (8.9%)	10 (9.1%)	0.819
		+	141 (91.0%)	41 (91.1%)	100 (90.9%)	
CK5/6	39.2%	−	69 (46.6%)	11 (32.4%)	58 (50.9%)	0.057
		+	79 (53.4%)	23 (67.6%)	56 (49.1%)	
TRPS1	31.5%	−	113 (95.0%)	29 (93.5%)	84 (95.5%)	0.677
		+	6 (5.0%)	2 (6.5%)	4 (4.5%)	
CDX2	25.7%	−	95 (97.9%)	25 (100.0%)	70 (97.2%)	0.400
		+	2 (2.1%)	0 (0.0%)	2 (2.8%)	
INSM1	25.9%	−	97 (99.0%)	25 (96.2%)	72 (100.0%)	0.094
		+	1 (1.0%)	1 (3.8%)	0 (0.0%)	
P16	36.0%	−	51 (37.5%)	16 (53.3%)	35 (33.0%)	0.042
		+	85 (62.5%)	14 (46.7%)	71 (67.0%)	
P63	54.8%	−	9 (4.3%)	5 (8.1%)	4 (2.8%)	0.086
		+	198 (95.7%)	57 (91.9%)	141 (97.2%)	
Uroplakin3	50.8%	−	102 (53.1%)	38 (69.1%)	64 (46.7%)	0.005
		+	90 (46.9%)	17 (30.9%)	73 (53.3%)	
AR	49.7%	−	114 (60.6%)	36 (66.7%)	78 (58.2%)	0.283
		+	74 (39.4%)	18 (33.3%)	56 (41.8%)	
Ki‐67	95.2%	%	0.40 (0.40)	0.30 (0.50)	0.40 (0.35)	0.013^a^

^a^ Tested with Wilcoxon rank‐sum test.

## 4 Discussion

To our knowledge, this is the largest study to date examining the clinicopathological features across the full spectrum of HER2 expression (negative, low, and overexpression) in Chinese UC patients. By incorporating the HER2‐negative group and analyzing a panel of additional IHC markers, our findings refine the understanding of UC heterogeneity and provide crucial data to inform patient selection for HER2‐targeted ADCs.

When comparing our findings to Western cohorts, the HER2 expression rates observed in our study present some notable differences. In our Chinese cohort, we identified a high HER2 overexpression rate (IHC 2+/3+) of 58.4% and a HER2‐low (IHC 1+) rate of 23.0%. In contrast, studies from European and North American cohorts report highly variable HER2 expression rates. A comprehensive systematic review highlighted this heterogeneity, showing that HER2 overexpression (IHC 2+/3+ or FISH+) ranged from 6.7% to 83% across different studies, underscoring that differences in testing methods and scoring criteria are major contributors to this variability [11]. For example, a large genomics‐based study of Western patients with metastatic urothelial carcinoma found the incidence of ERBB2 amplification—a molecular surrogate for HER2 overexpression—to be approximately 15% [12], a figure substantially lower than the overexpression rate detected by IHC in our study. Another recent Western cohort reported rates of 13% for HER2 overexpression (IHC 3+) and 48% for HER2‐low expression (IHC 1+/2+) [13]. These discrepancies may stem from multiple factors, including the use of different IHC antibodies, variations in scoring criteria (e.g., applying breast vs. gastric cancer guidelines), and potential underlying ethnic or biological differences. Therefore, our study provides valuable, population‐specific baseline data on the HER2 expression landscape in Chinese patients with urothelial carcinoma.

Our study confirms that HER2 is frequently expressed in UC, with 81.4% of patients showing some level of expression (IHC ≥ 1+). The strong, progressive association between higher HER2 expression and adverse features—such as bladder origin, infiltrative growth, and high‐grade histology—highlights its role in tumor biology. The finding that HER2 overexpression is more common in bladder tumors than in UTUC aligns with previous reports and has been recently corroborated in a large cohort study presented at ASCO, which also found HER2 3+ expression to be significantly tied to bladder primary origin [14]. With its large sample size, the study provides robust evidence for the link between HER2 status and primary site in a Chinese population. However, we did not find a significant association between HER2 status and the presence of distant metastasis at diagnosis. Further research is needed to explore the relationship between HER2 expression and patterns of metastatic spread, a topic highlighted by Zhu et al., who discussed the interplay between HER2 and other molecular drivers in metastatic UC [15].

A key strength of our study is the detailed characterization of the HER2‐low (IHC 1+) population, which comprised 23.0% of our cohort. This group is of increasing clinical importance due to the demonstrated efficacy of novel ADCs in HER2‐low tumors across different cancers, including UC [15]. Our analysis reveals that HER2‐low UC occupies an intermediate position in terms of clinicopathological risk factors between HER2‐negative and HER2‐overexpressing tumors. This distinction is vital for clinical trial design and future treatment guidelines. While our multivariable model identified predictors for HER2 overexpression versus low expression, the modest AUC of 0.64 suggests that clinical features alone are insufficient to reliably predict HER2 status. This underscores the indispensable role of IHC testing for accurate patient stratification.

This study reveals a strong association between the level of HER2 expression and the primary tumor site, with higher HER2 expression being progressively more common in tumors originating from the bladder (IHC 0: 62.2% vs. IHC 1+: 72.7% vs. IHC 2+/3+: 82.6%). This finding is consistent with and corroborated by recent data from Black et al. [14], who reported in a cohort of 209 UC patients that HER2 3+ status was significantly associated with a bladder primary origin (89%) compared to lower HER2 expression levels (70%–74%). This consistency across different populations reinforces the biological link between high HER2 expression and a bladder‐specific tumor phenotype.

In contrast, when examining metastatic behavior, our study did not find a significant association between HER2 expression level and the presence of distant metastasis at diagnosis. It is plausible, however, that HER2 status may not influence the overall incidence of metastasis but rather the specific pattern of organ involvement, a concept known as “organotropism.” For instance, a study by Alhalabi et al. [16] on metastatic UC demonstrated that a distinct molecular subtype defined by MTAP deficiency was associated with a significantly higher rate of visceral metastases, suggesting that specific genomic alterations can dictate metastatic sites. Therefore, while our data do not link HER2 to the overall risk of metastasis, future studies should investigate whether HER2 expression levels—particularly HER2‐low versus HER2‐high—correlate with specific patterns of metastatic spread (e.g., visceral, bone, or lymph node).

Our ancillary IHC analysis provides further insight into UC biology. The positive correlation between HER2 expression and markers like CK20 and Ki‐67 suggests a link to luminal‐like differentiation and higher proliferative activity. Conversely, the inverse relationships with Uroplakin 3 and P16 could indicate distinct underlying molecular pathways that warrant further investigation.

This study has several limitations. First, the lack of centralized pathology review and the use of different IHC testing protocols across the eight centers is a key concern. Although all centers adhered to a national consensus guideline, variability in assay sensitivity and interpretation could have influenced the results. Future studies should mandate centralized review to ensure consistency. Second, the retrospective design may introduce selection bias. Third, the ancillary IHC marker data were available for only a subset of patients from two centers, which limits the generalizability of these specific findings. Finally, our study did not have sufficient data to analyze the correlation between HER2 status and the specific sites of metastasis.

In conclusion, our findings emphasize the importance of developing personalized treatment strategies for UC patients based on the full spectrum of HER2 expression. Future research should focus on prospective, multicenter studies with centralized pathology review to validate these findings. Investigating the molecular mechanisms underlying different HER2 expression levels and their interplay with other biomarkers will be crucial for uncovering novel therapeutic targets and optimizing the use of powerful new agents like HER2‐targeted ADCs.

## 5 Conclusion

This large‐scale study delineates the distinct clinicopathological profiles of HER2‐negative, HER2‐low, and HER2‐overexpressing UC in a Chinese population. We identified significant associations between HER2 status and primary tumor site, infiltrative growth, and histologic grade, alongside novel correlations with other IHC markers. These findings provide critical evidence for tailoring HER2‐targeted therapies, particularly ADCs, and support more refined, personalized treatment strategies for UC patients in this population.

## Author Contributions

**Shanshan Wang:** conception and design; data acquisition; drafting the manuscript; statistical analysis; **Li Yang:** data acquisition; critical revision of the manuscript for scientific and factual content; statistical analysis; **Fan Cheng:** data acquisition; critical revision of the manuscript for scientific and factual content; statistical analysis; **Tiejun Yang:** data acquisition; critical revision of the manuscript for scientific and factual content; statistical analysis; **Xiaoping Zhang:** data acquisition; critical revision of the manuscript for scientific and factual content; statistical analysis; **Zhixian Yu:** data acquisition; critical revision of the manuscript for scientific and factual content; statistical analysis; **Qingyun Zhang:** data acquisition; critical revision of the manuscript for scientific and factual content; statistical analysis; **Yong Yang:** data acquisition; critical revision of the manuscript for scientific and factual content; statistical analysis; **Dingwei Ye:** conception and design; supervision; critical revision of the manuscript for scientific and factual content.

## Ethics Statement

This study was approved by the Ethics Committee of Fudan University Shanghai Cancer Center (Approval No.: 2301268–12); the principles of the Helsinki Declaration were followed. Informed consent was waived by the Ethics Committee of Fudan University Shanghai Cancer Center.

## Conflicts of Interest

The authors declare no conflicts of interest.

## Supporting information


**Data S1:** cam471289‐sup‐0001‐Supplementary Tables.docx.

## Data Availability

The data that support the findings of this study are available from the corresponding author upon reasonable request.
