# Supplementary material for: Distinct Clinicopathological Features of HER2‐Negative, HER2‐Low, and HER2‐Overexpressing Urothelial Carcinoma in a Large Chinese Cohort
Source: Cancer Med. 2025 Oct 10;14(19):e71289. doi: 10.1002/cam4.71289 (PMC12512340; doi:10.1002/cam4.71289)
Supplement: Supplementary file 1 — Data S1: cam471289‐sup‐0001‐Supplementary Tables.docx. [file CAM4-14-e71289-s001.docx]

**Supplementary Tables**

Supplementary Table S1. Multiple positive patterns (Top 10) of IHC test in UC patients

| **Positive markers** | **n** | **%** |
| --- | --- | --- |
| CK7 + CK20 + CK5/6 + P63 + Uroplakin3 | 31 | 8.2 |
| CK7 + CK20 + CK5/6 + P16 | 30 | 7.9 |
| CK7 + CK20 + CK5/6 + P63 + Uroplakin3 + AR | 27 | 7.1 |
| CK7 + CK20 + CK5/6 + P63 | 26 | 6.9 |
| CK7 + CK20 + GATA3 + CK5/6 + P16 | 25 | 6.6 |
| CK7 + CK20 + CK5/6 + P63 + AR | 18 | 4.8 |
| CK7 + CK20 + CK5/6 | 17 | 4.5 |
| CK20 + P63 | 17 | 4.5 |
| CK20 + CK5/6 + P63 | 17 | 4.5 |
| CK7 + CK20 + GATA3 + CK5/6 | 14 | 3.7 |
